# Zn Biofortification of Dutch Cucumbers with Chemically Modified Spent Coffee Grounds: Zn Enrichment and Nutritional Implications

**DOI:** 10.3390/foods13081146

**Published:** 2024-04-09

**Authors:** Beatriz Navajas-Porras, Ana Cervera-Mata, Alejandro Fernández-Arteaga, Adriana Delgado-Osorio, Miguel Navarro-Moreno, Daniel Hinojosa-Nogueira, Silvia Pastoriza, Gabriel Delgado, Miguel Navarro-Alarcón, José Ángel Rufián-Henares

**Affiliations:** 1 Departamento de Nutrición y Bromatología, Instituto de Nutrición y Tecnología de Alimentos, Centro de Investigación Biomédica, Universidad de Granada, 18011 Granada, Spain; beatriznavajas@ugr.es (B.N.-P.); adrianadelgado@ugr.es (A.D.-O.); miguelnav@correo.ugr.es (M.N.-M.); dhinojosa@ugr.es (D.H.-N.); spdelacueva@ugr.es (S.P.); nalarcon@ugr.es (M.N.-A.); 2 Department of Soil Science and Agricultural Chemistry, Faculty of Pharmacy, University of Granada, 18011 Granada, Spain; anacervera@ugr.es (A.C.-M.); gdelgado@ugr.es (G.D.); 3 Department of Chemical Engineering, University of Granada, 18071 Granada, Spain; jandro@ugr.es; 4 Instituto de Investigación Biosanitaria Ibs.Granada, Universidad de Granada, 18014 Granada, Spain

**Keywords:** cucumber, Zn, spent coffee grounds (SCGs), hydrochar, in vitro digestion, in vitro fermentation, antioxidant capacity, short-chain fatty acids

## Abstract

Spent coffee grounds (SCGs) are a food waste with a large generation around the world. However, their utilization as a soil organic amendment is difficult due to their phytotoxic effect. In the present work, the impact of agronomic biofortification on Dutch cucumbers was studied by using different chemically modified SCGs, analyzing their effects on Zn content, the release of antioxidant capacity and the production of short-chain fatty acids after in vitro digestion–fermentation. The results indicated variations in the Zn content and chemical composition of cucumbers according to the treatment groups. The functionalized with Zn and activated SCGs were able to increase Zn levels in cucumbers. Meanwhile, the activated hydrochar obtained at 160 °C and the activated and functionalized with Zn SCGs showed the highest Zn supply per serving. Differences in the antioxidant capacity and short-chain fatty acid production were observed between the groups. It is concluded that the growing conditions and the presence of Zn may significantly influence the contribution of these cucumbers to the dietary intake of nutrients and antioxidants, which could have important implications for human health and nutrition.

## 1. Introduction

Spent coffee grounds (SCGs) are the main by-product obtained during coffee brewing, mainly in coffee shops, with a worldwide production of around 15 million tons/year [1]. SCGs have a positive impact on soil quality, so that they can improve crop growth in the short term by increasing organic matter levels, soil fertility and carbon stock [2]. However, some compounds present in the dregs can be toxic and can reduce microbial diversity when they accumulate, so their application should be moderate. As an alternative, biochar is being used, which comprises stable carbonaceous materials that can remain in the soil for hundreds of years, improving crop fertility and plant growth [3]. It also has environmental benefits, as it can reduce greenhouse gas emissions and promotes the sustainable management of organic waste. Hydrochar, on the other hand, is similar to biochar, but is produced from hydrolysis instead of pyrolysis [4].

To improve the physical, chemical and biological properties of crops and soil quality, organic amendment (organic matter added to croplands) is carried out. This practice is commonly used for its positive influence on soil’s biodiversity and biochemical parameters and provision of essential nutrients for crop growth, among others [5]. Despite this, organic amendment also has some disadvantages, as the quality and composition of amendments may vary, being limited in certain regions, or supplying nutrients more slowly than other fertilizers [6]. Due to this, alternatives to organic matter have been proposed for use in crop improvement.

In the context of organic amendment, agronomic biofortification is a procedure that aims to increase the content of essential nutrients in crops and combat malnutrition in populations that depend on crops as their main food source [7]. Biofortification can increase the mineral content in the edible portion of the crop, improving the nutritional value of food. In addition, micronutrient fertilization can have a positive impact on other nutritional parameters of crops, such as the protein content, amino acids, phenolic compounds, chlorophyll, carotenoids and essential oils [8]. One of the most used elements for agronomic biofortification is Zn, since it is an essential mineral for humans as well as plants [7,9].

Cucumber (*Cucumis sativus* L.) belongs to the Cucurbitaceae family and is one of the most cultivated vegetables in the world. Despite its high water content, it contains bioactive compounds such as tannins, terpenoids, saponins, cardiac glycosides and dietary fiber that provide remarkable antioxidant capacity [10]. In this sense, dietary fiber (and attached phenolic compounds) is poorly digested by human beings, being transformed by the gut microbiota in the colon into simpler metabolites with enhanced absorption and bioactivity [11]. The main metabolites of dietary fiber are short-chain fatty acids (SCFAs) such as acetic, propionic and butyric acids, with demonstrated health properties [12]. In addition, the gut microbiota also releases antioxidant compounds from the food matrix [13].

Considering all the above stated information, the aim of this study was to evaluate the effect of agronomic biofortification of Dutch cucumbers by using different soil treatments based on SCGs: activated spent coffee grounds (ASCGs), Zn-functionalized and activated coffee grounds (ASCG-Zn), activated hydrochar of SCGs at 160 °C (AH160) and Zn-functionalized and activated SCG hydrochar (AH160-Zn). Modifications in the Zn content, as well as the release of antioxidant capacity and production of SCFAs after human digestion–fermentation, were assessed. Finally, the contribution of the consumption of cucumbers to the daily intake of Zn and polyphenols in the Spanish diet was also calculated. It is important to highlight that mineral biofortification is an innovative and relevant strategy due to the importance of minerals in health, the prevalence of mineral deficiencies in the diet and its potential as a sustainable agronomic solution [14]. Thus, the novelty of this paper compared to existing ones relies on three aspects: (i) the use of a food waste (spent coffee grounds) as a source of bio-chelates for mineral biofortification; (ii) the study of the effect of Zn biofortification on other nutritional parameters (such as total phenolic content and release of antioxidant capacity and short-chain fatty acids after in vitro digestion–fermentation); and (iii) the evaluation of the contribution to daily intake of the biofortified cucumbers on Zn and polyphenols intake. 

## 2. Materials and Methods

### 2.1. SCGs Bio-Products 

SCGs were acquired from the cafeteria of the Faculty of Pharmacy (University of Granada). They were spread into a thin layer and dried at room temperature (18–21 °C) to remove residual moisture for 7 days. Four soil treatments (based on SCGs) were obtained applying the same methodology described by Lara-Ramos et al. [3]: SCGs hydrochar at 160 °C (H160), activated SCGs (ASCGs) and hydrochar (AH160), and their corresponding Zn bio-chelates activated and functionalized (ASCG-Zn, AH160-Zn). The Zn content of the bio-chelates was determined using mineralization with HNO_3_ (65%) and H_2_O_2_ at 185 °C for 20 min in a microwave digestor (Multiwave 5000 with Rotor 24HVT50, Anton Paar GmbH, Graz, Austria). Mineral ions in the previous extracts were measured using inductively coupled plasma mass spectrometry (ICP-MS/MS; Agilent 8900 Triple Quadrupole ICP-MS/MS, Agilent Technologies Inc., Santa Clara, CA, USA). The final Zn content of the bio-products used in this study, expressed in mg/kg, was as follows: ASCGs: 6.5 ± 0.5; AH160: 15.5 ± 0.1; ASCG-Zn: 11,123 ± 242; AH160-Zn: 11,799 ± 241.

### 2.2. Greenhouse Experiment 

The experiment was conducted in a greenhouse of the “Fundación Cajamar” Experimental Station located in Almería, Spain (36°47′23″ N, 2°43′13″ W; 155 m a.s.l.) from October 2022 to January 2023. The site is located in a semi-arid subtropical Mediterranean climate with an average minimum temperature of 12.2 °C and an average maximum temperature of 16.1 °C corresponding to the aforementioned period; with these oscillations in external temperature the mean temperature in the greenhouse was 23.0 °C (ranging from 19.2 to 25.6 °C). The average annual precipitation is 220.5 mm while the mean solar radiation is 4576 Wh/m^2^ day. The plot used had dimensions of 30 m long and 8.5 m wide, with a total useful area of 255 m^2^.

The soil of the experiment was previously characterized by the staff of the experimental station. According to the internal report the soil has the following properties: sand 69.79%, silt 17.57%, clay 12.64%, pH 7.4, electrical conductivity at 25 °C (EC25) 2.05 dS/m, organic carbon (OC) 1.35%, carbonates as CaCO_3_ 29.2%, C/N 8.06, total N 0.17%, available P 318.1 mg/kg. 

The assayed bio-products at doses of 0.2% were as follows: ASCGs, AH160, ASCG-Zn, and AH160-Zn. These bio-products were added at 0.2%, since this is the proper dosage to avoid phytotoxicity for plants [3]. The 0.2% dosage corresponds to different amounts of Zn added (mg/kg) with the bio-chelates: ASCG: 0.011; AH160: 0.026; ASCG-Zn: 19; AH160-Zn: 20. Two controls were established: soil without any bio-product (Control) and a commercial Zn chelate at a concentration of 10 mg/kg soil (Control-Zn). The commercial chelate Zn ethylenediaminetetraacetate (EDTA-Zn, 14% *w*:*w*) was supplied by Trade Corporation International, S.A.U. As a summary, the study involved a total of six treatments (Table 1). The experiment was set up with four replicates in split-plots design. Two plots were used, each plot containing one replicate per treatment randomly distributed, composed of 4 plants. Overall, each treatment consisted of 8 plants (*n* = 8) per plot.

The planting points were distributed as follows: 1.5 m between crop lines and 0.5 m between plants within each crop line, meaning a planting density of 1.33 plants/m^2^. For the placement of the bio-products, a 20 cm deep × 15 cm diameter hole, equivalent to 4 kg of soil, was made in each planting point. The soil of all holes corresponding to the same treatment was extracted and homogenized for 5 min with the corresponding bio-product in a concrete mixer. The holes were then individually re-filled with the soil–bio-product mixture. The holes of the controls had the same soil treatment. The commercial chelate was dissolved in distilled water and poured at the surface. 

Cucumber seedlings (*Cucumis sativus* L. var. ‘Almería’ cv. ‘Huracán’) that were 21 days old were acquired from the commercial greenhouse Acrena S.A.T 251 (Almería, Spain). One seedling per planting point was planted in 8 cm soil depth after 6 days of the soil preparation.

### 2.3. Crop Maintenance 

The experiment was managed organically, using fertilizers authorized for organic cultivation by applying the drip irrigation method. Each row of cucumber was irrigated using a polyethylene pipe. The emitters were spaced at a 50 cm interval with a daily irrigation rate of 3 L/(h.m^2^) from 8:00 till 11:00. For the first 15 days after transplant, only water was used for irrigation; from day 16 till the end of the experiment the cultivation was irrigated with a nutrient solution containing 10 mM/L of N, 1.41 mM/L of P and 5.56 mM/L of K based on an organic fertilizer with a richness of 3.5% N, 2.5% P_2_O_5_ and 6.5% K_2_O diluted to a concentration of 3.2‰. The concentration of nitrate, potassium, calcium and sodium in the extracted soil solution was monitored biweekly using suction probes to maintain a nitrate concentration in a range of 3 to 10 mM/L and a K/Ca ratio between 0.5 and 1. These concentrations were measured using rapid analysis ionometers (LAQUAtwin, Horiba, Japan).

### 2.4. Plant Sampling and Processing

Cucumbers were harvested after 105 days of cultivation. Then, the fruits were cleaned, chopped and divided for antioxidant capacity of Zn analysis. Those samples for antioxidant capacity and total phenolic content were stored at −80 °C until in vitro digestion–fermentation. The samples for Zn analysis were dried in aluminum trays at room temperature for 48 h for water loss, then placed in an oven at 50 °C for 72 h and weighed again (dry weight). The dried material was milled and stored at room temperature until analysis.

### 2.5. Zn Determination

Cucumbers were harvested following a 105-day cultivation period. Subsequently, the fruits underwent cleaning, chopping and division for antioxidant capacity and Zn analysis. Samples designated for antioxidant capacity assessment were preserved at −80 °C until subjected to in vitro digestion–fermentation. Meanwhile, samples intended for Zn analysis underwent a drying process in aluminum trays at room temperature for 48 h to eliminate water content. They were then placed in an oven at 50 °C for 72 h to achieve a constant dry weight. The dried material was subsequently ground into powder and stored at room temperature till analysis.

For Zn determination, 0.200 g of the homogenized and dried sample was weighed using a precision balance (Ohaus, model PA224C, Europe GmbH, Greifensee, Switzerland) in borosilicate tubes. To these tubes, 3 mL of 69% HNO_3_ (TraceSELECT, Honeywell, Fluka, France), 0.5 mL of 30% H_2_O_2_ (Merck Suprapur, Darmstadt, Germany) and 0.5 mL of Milli-Q reagent-grade water were added. Prior to mineralization, 3 mL of an 11.5% HNO_3_ solution was added to the Teflon cups of the microwave digester, where the borosilicate tubes containing the samples were placed. After optimization of a suitable time-temperature program (20 min at a temperature range of 150 to 185 °C), mineralization of the samples was carried out.

Post-mineralization, the samples were diluted to 50 mL with Milli-Q reagent-grade water to prepare the analytical solution. Zn content in the final analytical dissolution was measured using ICP-MS/MS. Calibration curves were prepared using serial dilutions from a standard solution of 1000 mg/L of zinc in HNO_3_ at 1% (ppm; Merck; Darmstadt, Germany). The “Internal Standard Kit (Ge, Ir, Rh Sc; ISC Science, batch 20210712)” was utilized for correcting counts per second (CPS) for the analyzed atomic mass of Zn (^66^Zn) to that of ^72^Ge. Measurements were conducted using the linear calibration method and performed in triplicate for each sample.

Prior to Zn concentration determination using the ICP/MS technique, the analytical parameters of the procedure were validated. The limit of detection (LOD) for Zn was determined to be 0.42 µg/L. Accuracy and precision of the method were evaluated using reference standards certified in Zn, with recovery percentages ranging from 98.3 to 102.1% and coefficients of variation averaging 3.56%. These results demonstrate the suitability of the mineralization and analysis technique for measuring Zn in cucumber samples. The results are expressed as mg Zn/100 g of fresh weight.

To evaluate the efficiency of the different bio-products used, the Zn utilization efficiency (UE) was calculated according to Zhao et al. [15]:

UE (%) = (Uptake in treatment-uptake in control)/Micronutrient added × 100

### 2.6. In Vitro Digestion and Fermentation

Samples were subjected to in vitro gastrointestinal fermentation and in vitro fermentation in triplicate, following previously described protocols [16]. First, an oral phase was carried out by adding cucumbers to falcon tubes along with simulated salivary fluid (1:1, *w*/*v*) consisting of salts (KCl, KH_2_PO_4_, NaHCO_3_, NaCl, MgCl_2_(H_2_O)_6_, NH_4_(CO_3_)_2_, CaCl_2_(H_2_O)_2_ and HCl) and α-amylase (75 U/mL). The falcon tubes were kept for 2 min at 37 °C under oscillation.

Subsequently, the gastric phase was performed. For this, 5 mL of simulated gastric fluid was added, simulating the content of gastric juices in salts (same salts as in the oral phase) and pepsin (2000 U/mL). On this occasion, the mixture was kept at 37 °C for 120 min, at pH 3 and in oscillation. Finally, the intestinal phase was carried out, in which 10 mL of simulated intestinal fluid was added, simulating the content of intestinal juices in salts (same salts as in the oral and gastric phase), bile salts and enzymes (specifically 67.2 mg/mL pancreatin was used). The mixture was kept at 37 °C for 120 min in oscillation as in the gastric phase, but this time at pH 7. Once the intestinal phase was finished, the tubes were placed on ice and the enzymatic reactions were stopped. The tubes were then centrifuged for 10 min at 3500 rpm. The resulting solid pellet served as an in vitro fermentation substrate, and represents the undigested portion entering the large intestine. The supernatant, which represents the fraction available for absorption in the small intestine, was stored in 1 mL tubes at −80 °C until analysis.

In vitro fermentation was carried out using faecal samples from five healthy donors with an average Body Mass Index of 21.3 who had abstained from antibiotics during the three months prior to the trial. The eligibility criteria of the European project Stance4Health were used for healthy donors [17]. The informed consent document was signed by faecal donors. That form included all of the information of the study as well as the exclusion and inclusion criteria. The study was conducted according to the guidelines of the Declaration of Helsinki. It was approved by the Ethics Committee of the University of Granada (protocol code 1080/CEIH/2020). The faeces were pulled to reduce inter-individual variability. The fermentation was carried out for 20 h at 37 °C under agitation. After completion of the in vitro fermentation, the samples were placed on ice, as after digestion, to stop microbial reactions, and then centrifuged at 3500 rpm for 10 min. The supernatant, representing the fraction available for absorption in the large intestine, was stored at −80 °C until analysis. The solid pellet, representing the unfermented portion, was properly disposed of.

The result of the in vitro gastrointestinal digestion and fermentation was two fractions: the digestion supernatant, which corresponds to absorption in the small intestine, and the fermentation supernatant, which corresponds to absorption in the large intestine.

### 2.7. Antioxidant Tests

The antioxidant capacity of the supernatants obtained after digestion and fermentation were studied. The sum of the antioxidant capacity of both was considered the total antioxidant capacity [18].

#### 2.7.1. Trolox Equivalent Antioxidant Capacity Referred to Reducing Capacity (TEAC_FRAP_ Assay)

The procedure used to determine the ability of the samples to reduce ferric iron was based on that described by Benzie and Strain [19] and modified for use with a microplate reader (Cytation 5, Agilent Technologies Inc., Santa Clara, CA, USA). Briefly, 280 μL of freshly prepared FRAP reagent was combined with 20 μL of digestion or fermentation supernatant in a 96-well plate. The antioxidant response was observed for half an hour. Trolox was used to create a calibration curve at a concentration of 0.01 to 4 mg/mL. The results are expressed using mmol Trolox equivalent/kg fresh weight.

#### 2.7.2. Trolox Equivalent Antioxidant Capacity against DPPH Radicals (TEAC_DPPH_ Assay)

The technique was modified to work with a microplate reader (Cytation 5, Agilent Technologies Inc., Santa Clara, CA, USA) and followed the protocol of Yen and Chen [20]. Briefly, 280 μL of DPPH reagent was combined with 20 μL of digestion supernatant or fermentation supernatant in duplicate. To make this reagent, 7.4 mg DPPH per 100 mL methanol was used. The antioxidant response was observed for a full hour. Trolox was used to create a calibration curve, with concentrations ranging from 0.01 to 4 mg/mL. Results are given in mmol Trolox equivalent/kg fresh weight.

#### 2.7.3. Trolox Equivalent Antioxidant Capacity against ABTS+ Radicals (TEAC_ABTS_ Assay)

The ABTS assay was conducted following the method described by Ozgen et al. [21] with minor adjustments. A stock solution of 7 mM ABTS was mixed with 2.45 mM potassium persulfate to generate ABTS+, which was then left to incubate at room temperature for 12–16 h in the absence of light before utilization. The ABTS working solution, which remained stable for two days, was diluted with a mixture of ethanol and water (50:50) to achieve an absorbance of 0.70 ± 0.02 at 730 nm. After a 20 min incubation period, absorbance measurements were taken using a Cytation 5 microplate reader (Agilent Technologies Inc., Santa Clara, CA, USA). Trolox dilutions ranging from 0.15 to 1.15 mM were employed for calibration purposes. Results are reported in mmol Trolox equivalent/kg fresh weight. 

#### 2.7.4. Total Phenolic Content: Folin–Ciocalteu (FC) Assay

A microplate reader (Cytation 5, Agilent Technologies Inc., Santa Clara, CA, USA) was used to estimate the total phenolic content. The procedure of Singleton and Rossi [22] was followed, with some modifications. The FC assay was carried out. The results are expressed as gallic acid equivalents (GAE) per kg of fresh weight.

### 2.8. Short-Chain Fatty Acids Analysis

Following a previous study [23], the analysis of SCFAs was performed using ultra-high performance liquid chromatography (UHPLC). There was no need for sample pre-treatment prior to injection. SCFAs standards, prepared in the mobile phase at concentrations ranging from 5 to 10,000 ppm, were quickly created. The mobile phase was supplied at a flow rate of 0.250 mL/min and consisted of a mixture of two solutions. The mobile phase consisted of aqueous acetonitrile (1%) and ultrapure water (99%), both acidified with 1% formic acid. After fermentation, 1 mL of the supernatant was centrifuged, filtered through a 0.22 µm nylon filter and then transferred to a vial for UHPLC analysis. The UV-Vis photodiode array detector (PDA) was set at 210 nm and the column was a reversed-phase Accucore™ C18 (ThermoFisher Scientific, Waltham, MA, USA) with a particle size of 2.6 µm and 150 mm in length. The analysis was performed twice and the information shown represents the mean values of the millimolar (mM) concentration of each SCFA.

### 2.9. Calculations of Daily Antioxidant and Zn Intakes

The contribution of each cucumber group to the overall dietary intake of polyphenols and Zn was assessed individually according to treatment variations and plot distinctions. This assessment took into account several factors: (i) the average daily cucumber consumption in Spain and portion size [24]; (ii) the total phenolic content of the samples previously determined after the FC test; (iii) the Zn content within each cucumber group. In addition, the total phenolic and Zn content of each food was standardized to the typical serving size in Spain [25] and resembled previously documented findings on polyphenol [26] and Zn [27] intake.

### 2.10. Statistical Analysis

First, the normality of the samples was studied using the Shapiro–Wilk test. Then, a one-way analysis of variance (ANOVA) was performed for statistical differences between treatments which were assessed using Tukey’s test. Student’s *t*-test was also performed for graphical plots. The significance level was set at 95% (*p* < 0.05). Statistical analysis was carried out in triplicate and performed using control (non-bio-product) cucumbers as the reference group, as well as comparisons between all cucumber groups with different treatments. Statgraphics Plus, version 5.1, as well as SPSS 26.0 for Windows (IBM SPSS Inc., New York, NY, USA), was used.

## 3. Results 

### 3.1. Zn Content

**Figure 1:**
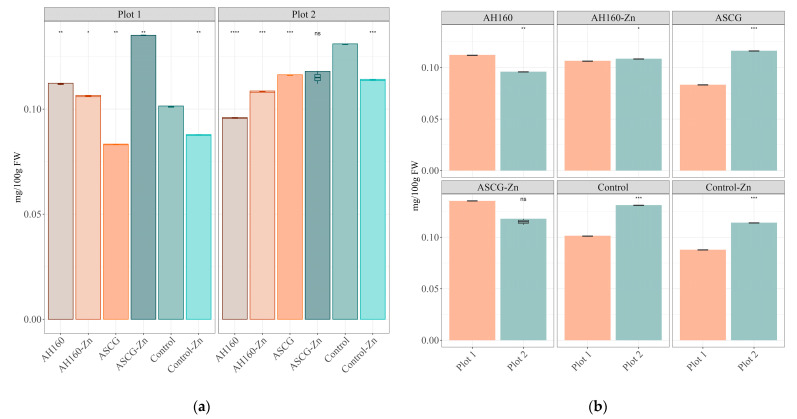
Differences in the Zn content of cucumbers. (**a**) Differences among treatment groups. Statistical analysis was performed using Student’s *t* test using the control group as the reference group. Statistical labels: *: *p* < 0.05, **: *p* < 0.01, ***: *p* < 0.001, ****: *p* < 0.0001, *ns*: not significant. (**b**) Differences between plots for each group. Statistical analysis was performed using Student’s *t*-test using Plot 1 as the reference group. Statistical labels: *: *p* < 0.05, **: *p* < 0.01, ***: *p* < 0.001, ns: not significant.

The results of Zn content for each group of cucumbers showed statistically significant differences (*p* < 0.05) in both plots (Figure 1a,b). First, comparisons were made with the control group (Figure 1a). In cucumbers from plot 1, the AH160, AH160-Zn and ASCG-Zn groups showed higher Zn levels (*p* < 0.05) than those of the control group. The treatment that increased to a greater extent the Zn content in cucumbers was ASCG-Zn, reaching levels of 0.13 mg/100 g fresh weight. The opposite was found for cucumbers of the ASCGs and Control-Zn groups, which reported lower Zn content (*p* < 0.05) than the control group. When comparisons were made between the different groups (Table 2), in addition to those already mentioned with the control group, statistically significant differences (*p* < 0.05) were found between all groups. 

In the case of plot 2, all cucumbers from the different groups (except the ASCG-Zn group) had lower Zn content (*p* < 0.05) than those from the control group, which reported higher Zn levels (reaching concentrations of 0.13 mg/100 g fresh weight). When comparisons were made between the different groups (Table 2), statistically significant differences (*p* < 0.05) were found between the AH160 group and all other groups. Almost the same was found for the AH160-Zn group: statistically significant differences between this group and all the other groups were found, except with control-Zn group.

Finally, differences between the two plots were studied for each cucumber group (Figure 1b). Statistically significant differences were found for all groups except for the ASCG-Zn group. Cucumbers from plot 2 had more Zn (*p* < 0.05) than those from plot 1 for the AH160-Zn, ASCGs, control and control-Zn groups, while the opposite was found for the AH160 group. 

### 3.2. Antioxidant Capacity

**Figure 2:**
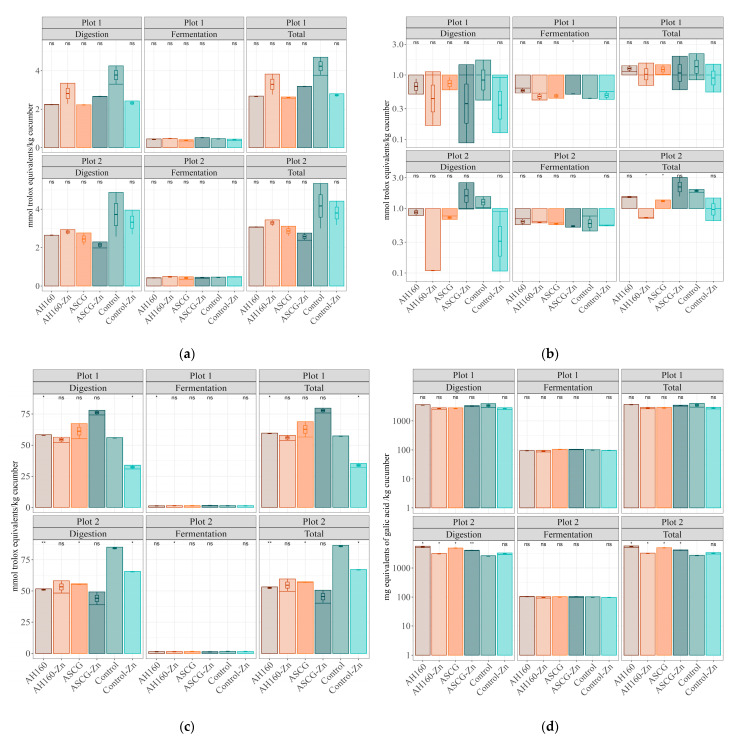
Antioxidant capacity of the different cucumber groups (obtained after in vitro digestion, in vitro fermentation and total antioxidant capacity). (**a**) The Trolox equivalent antioxidant capacity referred to reducing capacity (TEAC_FRAP_). (**b**) The Trolox equivalent antioxidant capacity against DPPH radicals (TEAC_DPPH_). (**c**) The Trolox equivalent antioxidant capacity against ABTS radicals (TEAC_ABTS_). (**d**) The total phenolic content (Folic–Ciocalteu). Results were log10 transformed to improve visualization. Statistical analysis was performed using Student’s *t* test using the control group as the reference group. Statistical labels: *: *p* < 0.05, **: *p* < 0.01, ns: not significant.

Regarding the antioxidant capacity released after in vitro digestion and fermentation, comparisons were made with those cucumbers belonging to the control group. For the FRAP method, no statistically significant differences were found (Figure 2a). In addition, comparisons were made between all cucumber groups taking into account the total antioxidant capacity. For neither of the two plots were statistically significant differences found between both groups (Table 2).

In the case of the DPPH method (Figure 2b), comparing all groups with respect to the control, statistically significant differences (*p* < 0.05) were found in the in vitro fermentation fraction for samples from plot 1, where ASCG-Zn cucumbers showed higher antioxidant capacity than those of the control group. In plot 2, statistically significant differences were found for total antioxidant capacity, where ASCGs and AH160-Zn cucumbers showed significantly (*p* < 0.05) lower antioxidant capacity than the control group. When studying the comparisons between the different groups (Table 2) there were no significant differences in their antioxidant capacity.

For the ABTS method, when comparisons were made with the control group (Figure 2c), significant differences were found for both factions (in vitro digestion, in vitro fermentation) and total antioxidant capacity. In plot 1, statistically significant differences (*p* < 0.05) were obtained between the AH160 cucumbers and the control group, as well as between the control-Zn and the control group (in the digestion fraction and total antioxidant capacity); the antioxidant capacity of AH160 was higher and that of the control-Zn group. In the in vitro fermentation fraction, the antioxidant capacity of the AH160 group was also significantly (*p* < 0.05) higher than that of the control group. On the other hand, for the samples from plot 2, the antioxidant capacity of the AH160, ASCGs and control-Zn groups were lower (*p* < 0.05) than that of the control group (for the in vitro digestion fraction and total antioxidant capacity). In the in vitro fermentation fraction, only the antioxidant capacity of the AH160-Zn group was significantly lower (*p* < 0.05) than that of the control group. When multiple comparisons (ANOVA) were performed among all cucumber groups (Table 2), statistically significant differences were also found. In addition to those already stated for plot 1 with respect to the control group, it was found that the antioxidant capacity of ASCG-Zn cucumbers was significantly higher than that of the AH160, AH160-Zn, ASCG and control-Zn groups (*p* < 0.05). On the other hand, the control-Zn group showed lower antioxidant capacity than AH160 and ASCG groups (*p* < 0.05). For plot 2, again the antioxidant capacity of the ASCG-Fe group was higher than that of the AH160 and control-Zn groups (*p* < 0.05).

Finally, for the FC method, no significant differences were found in plot 1 (Figure 2d) when comparisons were made with the control group. In plot 2, the antioxidant capacity of the AH160, AH160-Zn, ASCGs and ASCG-Zn groups was higher (*p* < 0.05) than that of the control group (in the in vitro fraction and total antioxidant capacity). In the in vitro fermentation fraction, no significant differences were found. In the ANOVA analysis (Table 2) no significant differences were found in plot 1. For plot 2, in addition to the aforementioned differences with respect to the control group, it was found a higher antioxidant capacity in AH160 cucumbers compared to the other groups (*p* < 0.05), except for the ASCG group (no differences were found). In addition, the antioxidant capacity of the AH160-Zn group was lower (*p* < 0.05) than that of the ASCG and ASCG-Zn groups. The control-Zn group had a lower antioxidant capacity than the ASCGs and ASCG-Zn groups.

#### Differences in Antioxidant Capacity in the In Vitro Digestion–Fermentation Fractions

**Figure 3:**
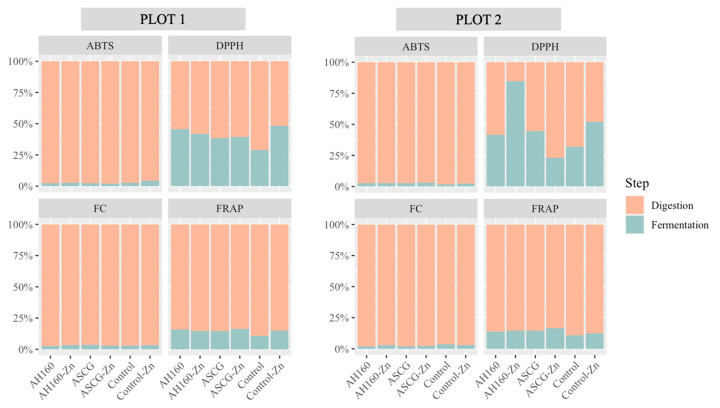
Contribution to the total antioxidant capacity (ABTS, DPPH, FC and FRAP) of each fraction in every group of cucumbers for both plots.

Figure 3 depicts the antioxidant capacity of both fractions obtained after in vitro digestion and fermentation. In general, the antioxidant capacity after in vitro fermentation decreased considerably compared to that obtained after in vitro digestion. This occurred for both ABTS and FC methods and in both plots. For the FRAP method, the fraction belonging to in vitro fermentation did not reach 25% for any of the treated groups of cucumbers, nor for any of the plots. In the case of the DPPH method, it was observed that the contribution to the total antioxidant capacity of the in vitro fermentation fraction was higher than for the other methods, although none of the treated cucumber groups reached 50% for plot 1; in addition, the AH160-Fe group exceeded 75% in plot 2. Therefore, in vitro digestion contributes more to the release of total antioxidant capacity.

### 3.3. Short-Chain Fatty Acids Produced after In Vitro Fermentation

**Figure 4:**
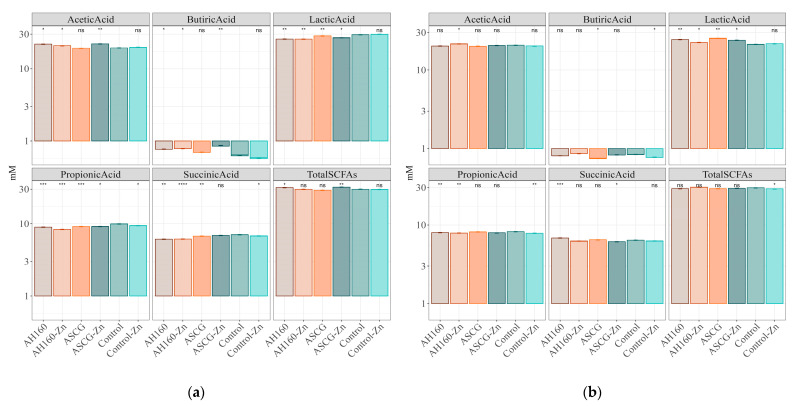
SCFAs produced after in vitro fermentation of cucumbers. (**a**) Plot 1. (**b**) Plot 2. Results were log10 transformed to improve visualization. Statistical analysis performed using Student’s *t* test (control group as reference). Statistical labels: *: *p* < 0.05, **: *p* < 0.01, ***: *p* < 0.001, ****: *p* < 0.0001, ns: not significant.

The production of SCFAs after in vitro fermentation of cucumbers from all groups was measured. First, comparisons were made between the different cucumber groups and the control, and then comparisons were made between all groups. The results of plot 1 (Figure 4a) showed that, for acetic acid, all cucumber groups were statistically significantly different (*p* < 0.05) to the control group, except the ASCGs and control-Zn groups, where no differences were found. For butyric acid, higher levels were produced after fermentation of AH160, AH160-Zn and ASCG-Zn cucumbers (*p* < 0.05) compared to the control group. For lactic and propionic acids, significant differences (*p* < 0.05) were found in the production of these short-chain fatty acids in all groups of cucumbers with respect to the control group (except for lactic acid production after control-Zn group fermentation). The same was true for succinic acid, although here, the ASCG-Zn group did not show significant differences with the control group. Finally, regarding the production of total SCFAs, statistically significant differences (*p* < 0.05) were found for AH160 and ASCG-Zn cucumber groups with the control group. Similar results were obtained for plot 2 (Figure 4b). When multiple comparisons (ANOVA) were made between all groups (Table 3), numerous statistically significant differences were found. Most of the differences were found for succinic acid production than for the other SCFAs. 

### 3.4. Influence of the Different Study Variables in Both Plots

**Figure 5:**
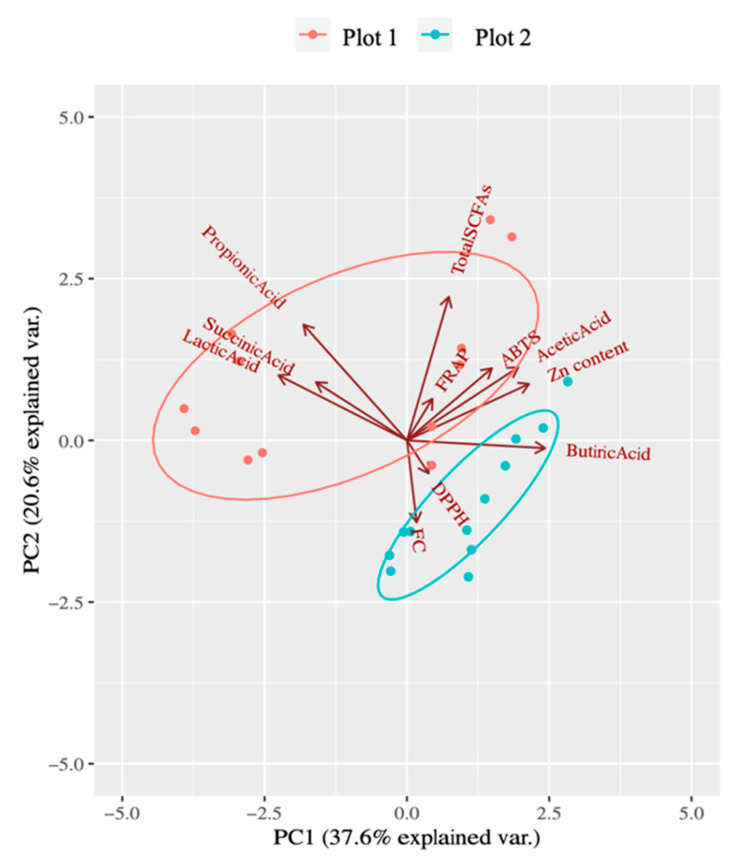
Principal component analysis (PCA) biplot chart. Total antioxidant capacity (FRAP, DPPH, ABTS and FC), short-chain fatty acids production and Zn content of the samples were taken into account. Arrows indicate in which plot the variable influence is higher.

In order to study the significance of the different variables studied in both plots, a principal component analysis was performed (Figure 5). Since the variables included in the analysis where very different (Zn content, antioxidant capacity and SCFAs) the variability explained for both plots was not very high (total 58.2%, 37.6 for PC1 and 20.6 for PC2). The samples from plot 1 showed higher contributions to the explained variability of lactic, succinic, propionic and acetic acids, total SCFAs, ABTS and FRAP. In the case of plot 2, DPPH, FC and butyric acid showed high contributions. It is well known that soil structure affects plant growth in many ways. The uptake of water and nutrients by plants can be limited by inadequate contact with the solid and liquid phases of the soil [28]. This could explain why such differences in soil could have a direct impact on the nutritional properties of cucumbers.

## 4. Discussion

In this research it has been tested if spent coffee grounds, a bio-waste with a toxic potential for plants that also contributes to CO_2_ release into the atmosphere [3,5], can be re-used after proper transformation to improve the nutritional value of vegetables by means of agronomic biofortification with Zn. In general, the bio-chelates that mostly increased the Zn content in cucumbers were the activated and functionalized hydrochar from SCGs in both plots (Figure 1a). To better understand the dynamics of Zn in this greenhouse experiment, the Zn utilization efficiency (UE) for cucumbers in relation to the total amount of added Zn was calculated. The following order was obtained in plot 1: AH160 (40.034 ± 0.132) > ASCG-Zn (0.174 ± 0.003) > AH160-Zn (0.024 ± 0.005) > control-Zn (−0.133 ± 0.008) > ASCGs (−162.857 ± 7.519). In the case of plot 2, the results for this parameter were as follows: ASCG-Zn (−0.081 ± 0.019) > AH160-Zn (−0.108 ± 0.003) > control-Fe (−0.169 ± 0.007) > AH160 (−129.716 ± 2.504) > ASCGs (−133.728 ± 0.140). If we compare these results with those in [3], which used activated and functionalized Zn hydrochar for growing lettuces, very similar trends were obtained. The activated and functionalized spent coffee grounds (ASCG-Zn) had a higher UE than the activated and functionalized hydrochar at 160 °C, both being higher than the commercial Zn-chelate. This is a positive outcome when comparing the bio-chelates with the control-Zn cucumbers. In fact, the Zn contained in the hydrochar particles [3] and activated and functionalized SCGs (ASCG-Zn) could be released into the medium over time and have a residual effect in subsequent cropping cycles [29]. On the other hand, the commercial chelate (control-Zn) had a lower utilization efficiency, which can be explained by the leaching of Zn over the cultivation period. The soil moisture was been maintained at a potential of −20 kPa (which is higher than field capacity, −33 kPa), indicating excess water and a continuous leaching process. According to Weil and Brady [30], Zn is a micronutrient that is less available under conditions of high soil leaching.

The aim of agronomic biofortification is to improve the nutritional value of selected cultivars; in our case, cucumbers enriched with Zn. Thus, once demonstrated that SCGs could be a source of bio-chelates with biofortification activity, the amount of Zn provided to the diet by each group of cucumbers was studied and compared with the European Zn reference intake for middle-aged men [27]. The AH160 and ASCG-Zn groups (Table 4) were the groups with the highest contribution of Zn per serving (AH160: 0.20 mg for plot 1 and 0.17 mg for plot 2; ASCG-Zn: 0.15 mg for plot 1 and 0.20 for plot 2) as well as when compared to the reference intakes. In contrast, the ASCGs group provided the lowest daily Zn levels to the diet per intake (and per serving) in plot 1, and the control group in plot 2, which is justified by the utilization efficiency data previously presented. The contribution of the Zn provided by cucumbers taking into account their daily intake in Spain was very low (from 0.06 to 0.09% of the daily needs). The percentages of contribution per serving were higher (from 1.39 to 2.25% of the daily Zn requirements) but still low, as most of the Zn in the diet comes from foods of animal origin [31]. 

Cucumbers are vegetable foods that can contribute to the daily intake of bioactive compounds such as polyphenols [31]. According to a previous study [26], the daily intake of phenolic compounds in Spain is 1171 mg gallic acid equivalents/person/day (assessed with the Folin–Ciocalteu assay). Of these, approximately 80% come from fruits, vegetables and cereals. In our study, Table 4 shows that the group of cucumbers with the lowest contribution to the daily intake of polyphenols was the control-Zn and AH160-Zn groups for plot 1 and the control group for plot 2. On the other hand, the cucumbers with the highest contribution to the daily intake of phenolic compounds were the AH160 group, reaching 546 (plot 1) and 822 (plot 2) mg/gallic acid/serving (Table 4). When the percentage of contribution to the daily intake of polyphenols was studied, it ranged from 1.39 to 2.13% when the mean daily consumption of cucumbers in Spain was taken into account. However, if a more realistic approach is used (the intake per serving), then the contribution will reach up to a 70.2% (Table 4). Finally, in the case of other antioxidant capacity detection methods, the ASCG-Zn group stood out as the one with the highest antioxidant capacity, for example, with the ABTS method in plot 1 and the DPPH method in plot 2 (Figure 2b,c). 

Another important fact to be mentioned is the higher antioxidant capacity released during digestion compared to that released after fermentation. This could be related with the high digestibility of cucumber, since most of the compounds with antioxidant capacity were released after in vitro digestion and were not available for metabolization by the colonic microbiota.

Other authors have published data on the antioxidant capacity of different vegetables. In particular, for cucumber the values were 0.7 and 0.4 mmol Trolox/Kg cucumber for TEAC_FRAP_ and TEAC_ABTS_, respectively [32]. If we compare these data with those presented in this article, our results are much higher, ranging from 33.8 to 85.8 mmol Trolox/Kg cucumber for TEAC_FRAP_, and from 2.6 to 4.2 and mmol Trolox/Kg cucumber for TEAC_ABT_. These differences come from the previous in vitro digestion and fermentation step applied to our samples, which increases the release of antioxidant species. Wojtunik-Kulesza et al. [33] argued that in vitro digestion can have a significant effect on the release of antioxidant compounds from foods because those antioxidant compounds present in foods undergo structural changes during digestion, affecting their antioxidant capacity. Other authors also argue [34] that during in vitro digestion there is an increase in the concentration of bioactive compounds, improving their bioavailability at target sites, thus enhancing their antioxidant properties.

Regarding SCFAs, these are health-promoting metabolites produced by gut microbes that feed on undigested nutrients such as dietary fiber [35]. The differences found in SCFAs production were numerous in both plots and are important to be discussed. Van Leeuwen and colleagues [36] studied the influence of soil on vine development and grape quality. They described how the soil had a great influence on plant production, nutritional status and grape composition, so that different soil types can provide unique conditions that affect both plant growth and vegetable and fruit production, as observed in our experiments with Dutch cucumbers.

## 5. Conclusions

In this work, it has been demonstrated that SCGs can be transformed into smart Zn bio-chelates. Therefore, SCGs can be re-used for the biofortification of Dutch cucumbers, also avoiding their contaminant properties when disposed in landfills. The cucumbers grown with spent coffee grounds functionalized with Zn (ASCG-Zn) had the highest Zn levels and contribution to the daily intake of Zn for plot 2, while in the case of plot 1 activated SCGs hydrochar (AH160) were those with the highest contribution to Zn intake; however, in general, the Zn contribution of cucumbers to the human diet was low. Cucumbers grown with SCGs hydrochar (AH160) showed the highest contribution to the daily intake of polyphenols; however, in terms of antioxidant capacity, statistically significant differences were hardly found. Diversity in SCFAs production was also observed between groups of cucumbers, showing that the groups had a different chemical composition. Both the Zn content and the chemical composition of cucumbers may vary significantly depending on the growing conditions, which in turn may affect the contribution of these cucumbers to the dietary intake of nutrients and antioxidants, which may have important implications for human health and nutrition.

## Figures and Tables

**Table 1:** Treatment details of the greenhouse experiment.

N°	Treatment	Description
1	Control	No bio-product
2	Control-Zn	Commercial chelate (EDTA-Zn, 14%)
3	ASCGs	Activated spent coffee grounds (SCGs)
4	AH160	Activated hydrochar obtained from SCGs at 160 °C
5	ASCG-Zn	Activated SCGs functionalized with Zn
6	AH160-Zn	Activated hydrochar obtained from SCGs at 160 °C functionalized with Zn

**Table 2:** Differences in Zn content and antioxidant capacity (FRAP, DPPH, ABTS and FC assays) between cucumber groups. Differences in the two plots were studied separately. Statistical analysis was performed using ANOVA and Tukey’s test. Statistical labels: *: *p* < 0.05, ns: not significant.

	Zn Content	FRAP Assay	DPPH Assay	ABTS Assay	FC Assay
**Plot 1**					
AH160-Zn/AH160	*	ns	ns	ns	ns
ASCGs/AH160	*	ns	ns	ns	ns
ASCG-Zn/AH160	*	ns	ns	*	ns
Control-Zn/AH160	*	ns	ns	*	ns
ASCGs/AH160-Zn	*	ns	ns	ns	ns
ASCG-Zn/AH160-Zn	*	ns	ns	*	ns
Control-Zn/AH160-Zn	*	ns	ns	*	ns
ASCG-Zn/ASCGs	*	ns	ns	*	ns
Control-Zn/ASCGs	*	ns	ns	*	ns
Control-Zn/ASCG-Zn	*	ns	ns	*	ns
	**Zn Content**	**FRAP Assay**	**DPPH Assay**	**ABTS Assay**	**FC Assay**
**Plot 2**					
AH160-Zn/AH160	*	ns	ns	ns	*
ASCGs/AH160	*	ns	ns	ns	ns
ASCG-Zn/AH160	*	ns	ns	ns	*
Control-Zn/AH160	*	ns	ns	ns	*
ASCGs/AH160-Zn	*	ns	ns	ns	*
ASCG-Zn/AH160-Zn	*	ns	ns	*	*
Control-Zn/AH160-Zn	ns	ns	ns	ns	ns
ASCG-Zn/ASCGs	ns	ns	ns	ns	ns
Control-Zn/ASCGs	ns	ns	ns	ns	*
Control-Zn/ASCG-Zn	ns	ns	ns	*	*

**Table 3:** Differences in short-chain fatty acids production between cucumber groups. Differences in the two plots were studied separately. Statistical analysis was performed using ANOVA and Tukey’s test. Statistical labels: *: *p* < 0.05, ns: not significant.

Plot 1	Acetic Acid	Butyric Acid	Lactic Acid	Propionic Acid	Succinic Acid	Total SCFAs
AH160-Zn/AH160	*	ns	ns	*	ns	*
ASCGs/AH160	*	*	*	*	*	*
ASCG-Zn/AH160	ns	*	*	*	*	*
Control-Zn/AH160	*	*	*	*	*	*
ASCGs/AH160-Zn	*	*	*	*	ns	*
ASCG-Zn/AH160-Zn	*	*	*	*	*	*
Control-Zn/AH160-Zn	*	*	*	*	*	ns
ASCG-Zn/ASCGs	*	*	*	ns	*	*
Control-Zn/ASCGs	*	*	*	*	ns	*
Control-Zn/ASCG-Zn	*	*	*	*	*	*
**Plot 2**	**Acetic Acid**	**Butyric Acid**	**Lactic Acid**	**Propionic Acid**	**Succinic Acid**	**Total SCFAs**
AH160-Zn/AH160	*	*	*	ns	*	*
ASCGs/AH160	ns	*	*	*	*	ns
ASCG-Zn/AH160	ns	ns	*	ns	*	ns
Control-Zn/AH160	ns	*	*	*	*	ns
ASCGs/AH160-Zn	*	*	*	*	*	*
ASCG-Zn/AH160-Zn	*	*	*	ns	ns	*
Control-Zn/AH160-Zn	*	*	*	ns	ns	*
ASCG-Zn/ASCGs	*	*	*	*	*	ns
Control-Zn/ASCGs	ns	ns	*	*	*	ns
Control-Zn/ASCG-Zn	ns	*	*	ns	*	*

**Table 4:** Contribution of cucumber consumption to daily antioxidant capacity and Zn intake in the Spanish diet.

Group	Analytical Assay	Zn/Daily Intake * (mg Zn/day)		Zn/Serving Intake ^†^ (mg Zn/Serving)		Mean Contribution to Daily Zn Intake Compared with Previous Data ^#^ (%)		Mean Contribution to Daily Zn per Serving Intake ^#^ (%)	
		Plot 1	Plot 2	Plot 1	Plot 2	Plot 1	Plot 2	Plot 1	Plot 2
Control	Zn content	0.01	0.01	0.17	0.14	0.07	0.06	1.87	1.60
Control-Zn	Zn content	0.01	0.01	0.16	0.16	0.07	0.07	1.77	1.81
ASCGs	Zn content	0.00	0.01	0.12	0.17	0.06	0.08	1.39	1.94
AH160	Zn content	0.01	0.01	0.20	0.17	0.09	0.08	2.25	1.92
ASCG-Zn	Zn content	0.01	0.01	0.15	0.20	0.07	0.09	1.68	2.18
AH160-Zn	Zn content	0.01	0.01	0.13	0.17	0.06	0.08	1.46	1.90
**Group**	**Analytical assay**	Polyphenols/daily intake * (mg gallic acid/day)		**Polyphenols/serving intake** **^†^ (mg gallic acid/serving)**		**Mean contribution to daily polyphenols intake compared with previous data** **^★^ (%)**		**Mean contribution to daily polyphenols intake per serving** **^★^ (%)**	
		**Plot 1**	**Plot 2**	**Plot 1**	**Plot 2**	**Plot 1**	**Plot 2**	**Plot 1**	**Plot 2**
Control	FC assay	21.1	16.3	529	407	1.81	1.39	45.1	34.7
Control-Zn	FC assay	16.7	19.3	417	484	1.42	1.65	35.6	41.3
ASCGs	FC assay	16.8	29.9	421	748	1.44	2.56	35.9	63.9
AH160	FC assay	21.8	32.9	546	822	1.87	2.81	46.6	70.2
ASCG-Zn	FC assay	20.3	24.9	506	622	1.73	2.13	43.2	53.2
AH160-Zn	FC assay	16.7	19.3	418	482	1.43	1.65	35.7	41.1

* Considering daily consumption for the Spanish population. **^†^** Considering the intake of 1 serving. ^#^ Considering dietary reference intakes [27]. ^★^ Considering previous data [26].

## Data Availability

The original contributions presented in the study are included in the article, further inquiries can be directed to the corresponding author.

## Abbreviations

SCG: Spent Coffee Grounds, TEAC: Trolox Equivalent Antioxidant Capacity, GAE: Gallic Acid Equivalents, FC: Folin-Ciocalteu, SCFAs: Short Chain Fatty Acids.
